# Research Note: Tracing pathways of entry and persistence of facultative pathogenic and antibiotic-resistant bacteria in a commercial broiler farm with substantial health problems

**DOI:** 10.1016/j.psj.2020.08.050

**Published:** 2020-09-03

**Authors:** Céline Heinemann, Caroline D. Leubner, Mykhailo Savin, Esther Sib, Ricarda M. Schmithausen, Julia Steinhoff-Wagner

**Affiliations:** ∗ Institute of Animal Science, Preventive Health Management, University of Bonn, 53115 Bonn, Germany; † Institute of Animal Science, Cold Chain Management, University of Bonn, 53115 Bonn, Germany; ‡ Institute of Hygiene and Public Health, University Hospital Bonn, 53105 Bonn, Germany

**Keywords:** poultry production, antibiotic resistance, water pipe, *Pseudomonas aeruginosa*, risk factor

## Abstract

On a commercial broiler farm with substantial health problems, shown by a reported loss rate of approx. 10% and regular antibiotic use, samples were taken at different locations in 2 barns, with the aim of analyzing possible entry routes and persistence of pathogens and antibiotic-resistant bacteria as well as revealing weak points in sanitation. Therefore, swab samples for biofilm and water samples from animal drinking water lines and the spray cooling system were taken twice immediately before restocking. In addition, swab samples from drain holes and air samples were collected. At restocking, hatchlings that died during transportation and chick paper were sampled. All samples were analyzed for the occurrence of *Pseudomonas aeruginosa*, total coliform count, and antibiotic-resistant bacteria, namely, methicillin-resistant *Staphylococcus aureus* (**MRSA**), *Escherichia coli*, *Klebsiella* spp., *Citrobacter* spp., *Enterobacter* spp., *Acinetobacter baumannii*, *P. aeruginosa*, and vancomycin-resistant *Enterococci* (**VRE**). No MRSA or VRE were detectable. In all samples from drinking water and sprinkler system pipes, *P. aeruginosa* was detectable; in most cases, antibiotic-resistant *P. aeruginosa* was also detected, with varying resistance profiles. Samples from the hatchlings and chick paper were contaminated with antibiotic-resistant *Enterobacter* spp., with resistance to piperacillin, fosfomycin, and the third-generation cephalosporins cefotaxime and ceftazidime. Therefore, the initial entry of antibiotic-resistant *Enterobacteriaceae* likely occurred via exposure at the hatchery, resulting in colonization of the chicks. Animals on the fattening farm were treated with colistin, amoxicillin, and lincomycin in the 3 production cycles before sampling. Owing to the frequent administration of several antibiotic classes during the fattening period via piped water in both barns, resistance of isolates from water pipes accumulated, showing additional resistance to chloramphenicol and frequently to ciprofloxacin and levofloxacin. To prevent the development of secondary diseases caused by the facultative pathogen *P. aeruginosa* in chicks with a weak immune status, the hygiene management for drinking water lines and the spray cooling system was changed. These changes resulted in an improvement in water line sanitation, shown by the absence of antibiotic-resistant bacteria and rare detection of *P. aeruginosa*.

## Introduction

Frequent administration of antibiotics in livestock farming remains a problem, even if the use as a growth promoter has been abandoned in the EU since 2006 and application in Germany has significantly decreased, provoked by the 16th Act to Amend the Medicinal Products Act (BMEL, 2019). In poultry, compared with that in other farm animal species, antibiotic usage remains high, reflected in a negligible decrease from 29.7 t in the second term of 2014 to 29.5 t in the second term of 2017 (BMEL, 2019). This lack of change is partly because health problems among broilers can rapidly lead to major losses because of the high total number of animals and high animal density, resulting in a high risk of infection. Therefore, hygiene in poultry production is essential for performance and animal health maintenance (Luyckx et al., 2015). Furthermore, a high standard of hygiene forms the basis for minimal antibiotic use (Gleeson and Collins, 2015). In the chicken-fattening sector, cleaning and disinfection are frequently outsourced to cleaning contractors, with increasing tendency. Sanitation by cleaning contractors often leads to better results, probably caused by better knowledge and professional equipment (Maertens et al., 2018). However, farmers are still responsible for cleaning details, such as drinking water and sprinkler system pipes, which are sometimes neglected or insufficient, perhaps because of lack of time or deficient knowledge. The time allocated for sanitation is usually limited by an unchangeable scheduled delivery of new hatchlings, which explains why corrective measures are mostly impossible if unexpected challenges, such as delays in delivery of new hatchlings or transport of broilers to abattoirs, occur during sanitation. In addition to proper hygiene management, the chicks themselves constitute an important factor for later health and performance. The aim of this study was to determine the critical points of the entry and persistence of facultative pathogenic and antibiotic-resistant bacteria on a broiler-fattening farm with substantial health problems after cleaning and disinfection.

## Materials and methods

### Farm Characteristics and Sample Collection

**Figure 1:**
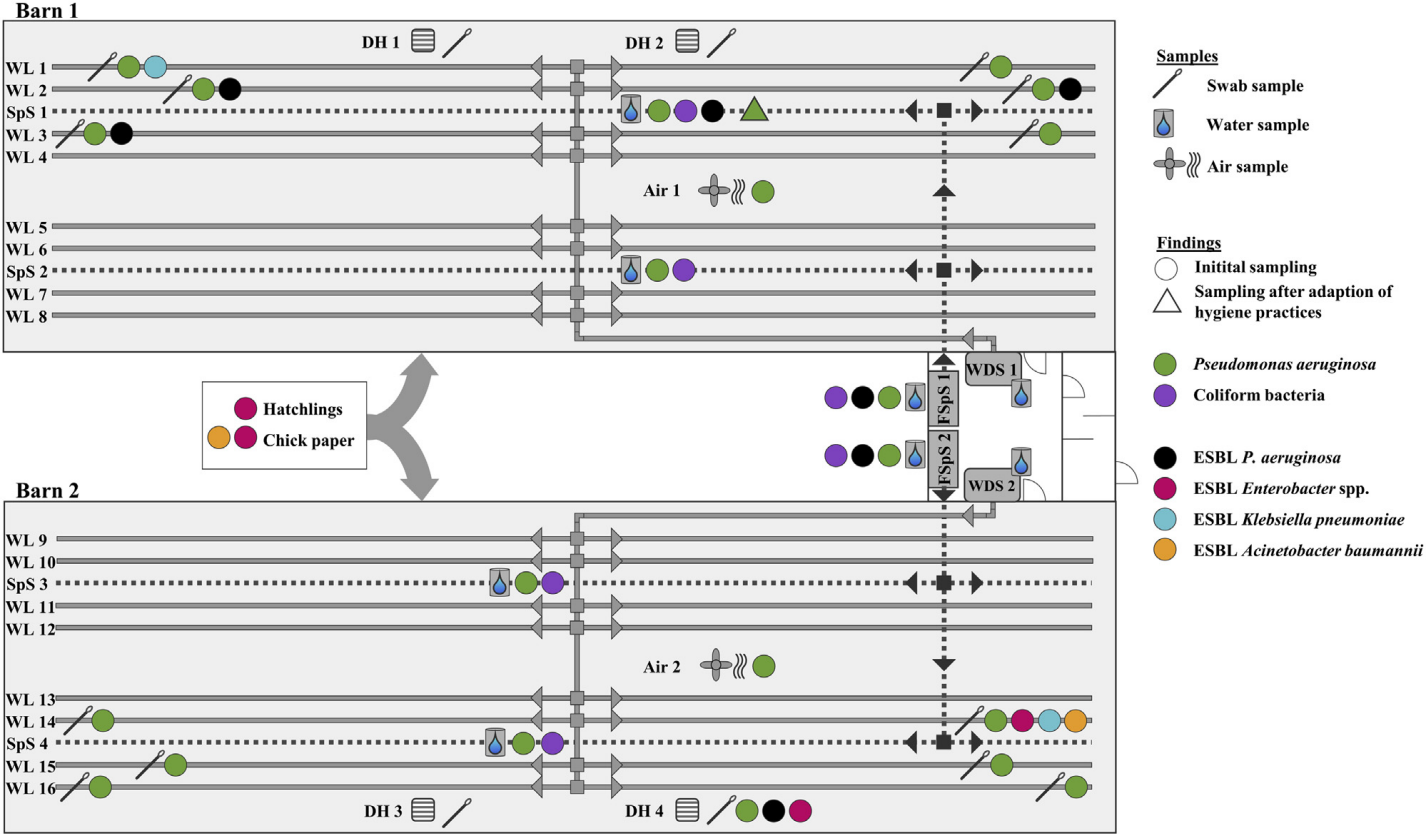
Swab samples were taken at 2 time points from animal drinking water lines (WL 1, 2, 3, 14, 15, and 16) from both sites and the pipes and drain holes (DHs 1, 2, 3, and 4) in both barns on the chicken-fattening farm. In the middle of both barns, air samples were collected (air 1 and 2). Water samples were taken from the sprinkler system (SpS 1 and 2), the filters of the sprinkler system (FSS 1 and 2), and the water dosing units (WDS 1 and 2) of both barns. In addition, cloacal and nasal swab samples from deceased hatchling and samples of the chick paper were analyzed. Abbreviation: ESBL, extended-spectrum beta-lactamase–producing bacteria.

Samples were taken at a commercial broiler farm located in North Rhine-Westphalia, Germany, with 79,000 fattening places for broilers distributed equally in 2 separate barns. Routine cleaning and disinfection of surfaces was carried out by a professional cleaning contractor. Cleaning was conducted with a high-pressure wash followed by disinfection with a disinfectant consisting of glutaraldehyde and quaternary ammonium compounds. Cleaning and disinfection of the drinking water system was performed by the farmer with an alkaline cleaner containing sodium hydroxide and disinfected with a combination of peracetic acid, acetic acid, and hydrogen peroxide. After disinfection, all the water of the drinking system was drained. During the fattening period, animal drinking water was disinfected continuously with a commercial product. The first sampling occurred 24 h before restocking, and the second sampling occurred immediately before restocking of the following production cycle to assess sanitation performance. Based on the results of the first sampling, hygiene measures were adapted as follows: reaction time for the detergent in the water drinking line was enhanced, with a subsequent thorough rinsing with fresh water. To avoid diluting the disinfectant effect, a drying time of 24 h was added before disinfection. Exposure time to disinfectants in the drinking water lines was enhanced from 2 to 12 h. The same procedure was implemented for the water sprinkler system. In addition, the filters of the sprinkler system were disassembled and immersed in disinfectant solution for 12 h. Improvement of hygiene status was assessed in the second sampling, by comparing the results from the first and second sampling. The farmer relayed the results of previously conducted antimicrobial susceptibility tests, from both production cycles before sampling, from a contract laboratory. The farmer reported that all broilers were treated in the last 3 mo before metaphylactic sampling with colistin, amoxicillin, and lincomycin to reduce animal losses. In total, 26 samples of swabs, water, and air were taken in both barns. The following areas were sampled in each barn: 6 animal drinking water lines, 2 water-sprinkler systems, one water-sprinkler system filter units, one dosing unit for medicinal products and nutritional supplements through drinking water lines, 2 drain holes, and one air sample per barn (Figure 1). In addition, pool swab samples from drinking cups and feeding troughs from both barns were collected. For swab samples, sterile flocked swabs with 1 mL of liquid Amies medium (eSwab, COPAN, Brescia, Italy) were used. Water samples were collected from areas of stagnating water in sterile tubes in 50-mL volumes. Collection of air samples was performed using a microbial air sampler (Coriolis micro, Bertin Technologies, Montigny Le Bretonneux, France) with 15 mL of sterile physiologic saline solution with 0.9% sodium chloride (Oxoid, Basingstoke, UK) and a flow rate of 250 L ∙ min^−1^ and 5-min sampling time, resulting in 1.25 m^3^ of sampled air. The system aspirates the air and deflects it in the saline solution. Particles >0.5 μm are deposited in the liquid and can be analyzed. Air samples were obtained in the center of the barns at a height of 50 cm above the ground. At the first restocking, nasal and cloacal swabs were collected from the 3 hatchlings that died during transport. Three samples of chick paper from transport boxes were taken. Samples were transported in insulated boxes to the laboratory and analyzed within 24 h.

### Microbiological Analysis

The Amies medium from swab samples, water samples, and air samples was directly analyzed without further dilution. A bulk sample of the 3 chick paper samples was created by weighing 5 g of each sample in blender bags with filter elements. In the filter bags, 145 mL of sterile physiologic saline solution was added. Samples were homogenized for 60 s with a bag mixer. All liquid samples were analyzed for total coliform count (Chromocult coliform agar, Merck, Darmstadt, Germany) as an indicator of fecal soiling via the pour plate technique. Plates were incubated at 37°C for 24 h. All blue and salmon-red colonies were counted as coliforms. For detection of *Pseudomonas aeruginosa*, cetrimide agar plates (Oxoid, Wesel, Germany) were used. Detection of antibiotic-resistant bacteria was performed with CHROMAgar plates (MAST Diagnostica, Reinfeld, Germany) for methicillin-resistant *Staphylococcus aureus* and for extended-spectrum beta-lactamase–producing bacteria (**ESBL**), namely, *Escherichia coli*, *Klebsiella* spp., *Citrobacter* spp., *Enterobacter* spp., *Acinetobacter baumannii*, *P. aeruginosa,* and vancomycin-resistant *Enterococci*. The abbreviation “ESBL” was used for colonies that grew on ESBL plates and with resistance to third-generation cephalosporins. The plates were inoculated with 1 mL, using the spread plate technique and incubated at 37°C for 24 h and 48 h according to manufacturer specifications. All cultural methods were conducted in duplicate. For testing susceptibility to antibiotics, suspicious colonies were subcultured on Columbia sheep blood (MAST Diagnostica) at 37°C for 24 h and identified by matrix-assisted laser desorption/ionization time of flight mass spectrometry with Myla software (bioMérieux, Marcy-ĺEtoile, France). Antibiotic susceptibility was tested via a microdilution assay using Micronaut-S MDR MRGN-Screening for gram-negative bacteria (MERLIN, Gesellschaft für mikrobiologische Diagnostika GmbH, Bornheim-Hersel, Germany). The results were interpreted according to the European Committee on Antimicrobial Susceptibility Testing clinical cutoff values for analyzing the resistance status of bacteria from the **ESKAPE** group (*Enterococcus* spp., *S. aureus*, *K. pneumoniae*, *A. baumannii, P. aeruginosa, Enterobacter* spp.) and *E. coli* of livestock origin against clinically important antimicrobials for humans.

## Results and discussion

**Figure 2:**
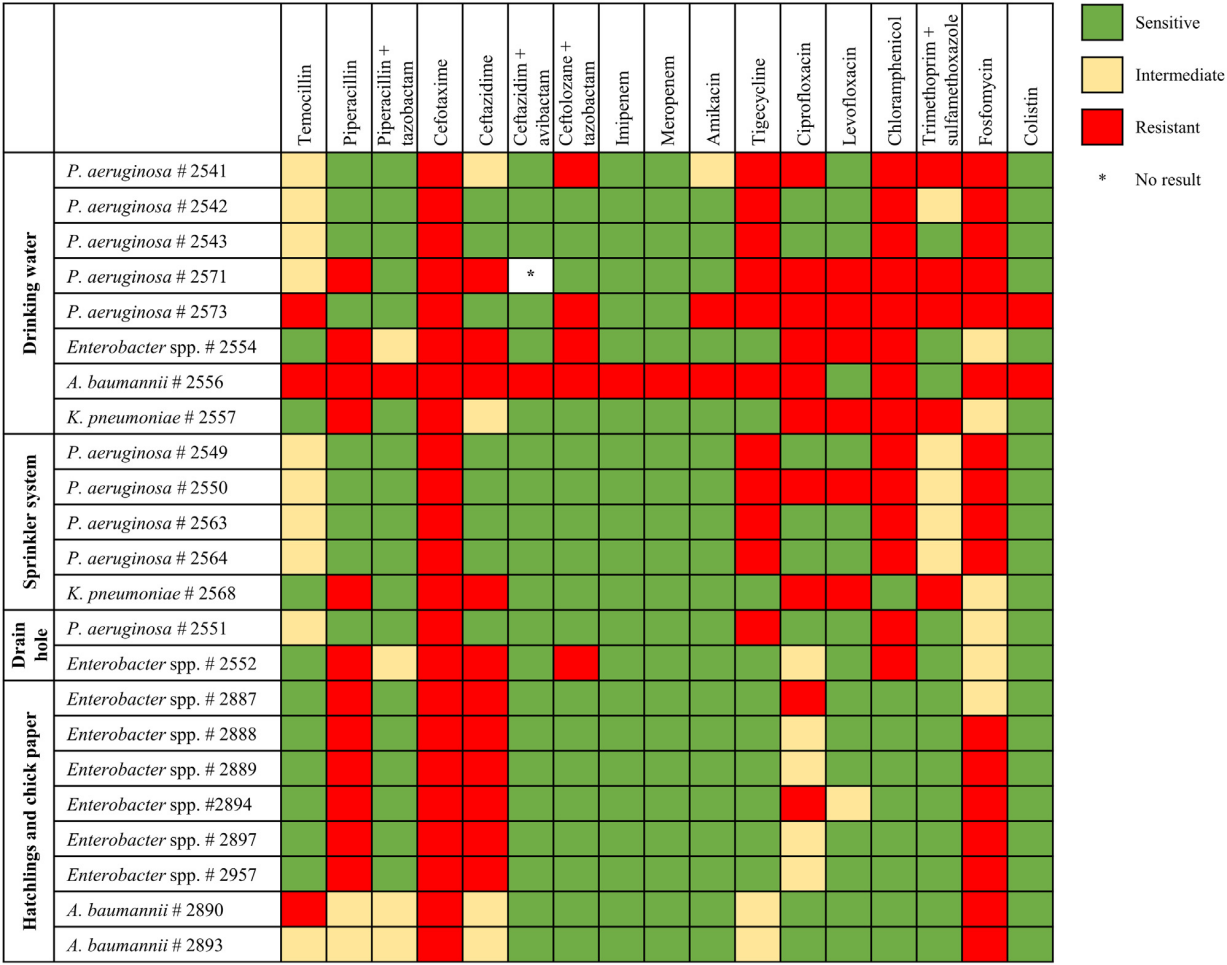
The results from antimicrobial susceptibility testing of the isolates from the chicken-fattening farm showed variation depending on the organism and the origin of the isolate.

For systematic investigation, a representative number of samples of biofilms and water were taken from drinking water lines (swabs), drain holes (swabs), air (collected as a bulk sample), sprinkler system (water), filters of the water sprinkler system (water), and the dosing unit for medicinal products (water) and were qualitatively analyzed for the occurrence of ESKAPE bacteria and *E. coli* in the first sampling (Figure 1). Both water samples from the dosing unit, as well as swab samples from the drinking cups and feeding troughs were negative for all tested parameters. This result demonstrated that the water was fed into the drinking system without bacterial contamination and the high quality of the cleaning contractor. On broiler farms, feeding troughs and drinking cups seem to be of minor importance as critical points in sanitation, unlike those on pig-fattening farms (Heinemann et al., 2020). Coliform bacteria were detectable in all water samples of the sprinkler system and filters of the sprinkler system. *Pseudomonas aeruginosa* was found in all swab samples from the drinking water system from both barns (n = 12), both air samples, and all water samples from the sprinkler system and the filter unit (n = 4) but in only one of the swab samples from the drain holes (n = 4) (Figure 1). For water and air samples, almost all samples exceeded the detection limit of 2.5 log_10_ cfu ⋅ ml^−1^. Phenotypical ESBL *P. aeruginosa* were detectable in 3 swab samples of the drinking water lines and one sample of the sprinkler system in barn 1, in samples of the filter of the sprinkler system from both barns, and in one swab sample of the drain hole in barn 2. The detection of phenotypical ESBL *Enterobacter* spp. (n = 2), *K. pneumoniae* (n = 2), and *A. baumannii* (n = 1) was less frequent. In all samples of the nasal swabs, in one cloacal swab of the hatchlings, and in the bulk sample of the chick paper, ESBL *Enterobacter* spp. were detected. In addition, ESBL *A. baumannii* was found in the chick paper sample (Figure 1). In none of the analyzed samples were methicillin-resistant *Staphylococcus aureus* or vancomycin-resistant *Enterococci* detectable. These results indicate that the entry of resistant *Enterobacter* spp. and *A. baumannii* occurred via colonized animals from the hatchery, whereas on the sampled farm, *P. aeruginosa* originated predominantly from the drinking water and sprinkler-system pipes. In previously performed antimicrobial susceptibility tests from a contract laboratory on 3 swab samples from yolk sacs immediately after hatching, *Enterococcus faecium* with resistance against trimethoprim with sulfadiazine, colistin, and tylosin and a reduced susceptibility toward enrofloxacin and penicillin was found. In addition, isolates of *Enterococcus faecalis* with resistance against trimethoprim/sulfadiazine, colistin, tylosin, and Linco-Spectin, which consists of lincomycin and spectinomycin, and a reduced susceptibility against penicillin were detected. In a sample from the pericardium of the hatchlings, *E. coli* was detected with resistance against amoxicillin, tylosin, and penicillin and a reduced susceptibility against Linco-Spectin. The farmer reported that administration of antimicrobials, such as colistin and lincomycin, resulted in insufficient recovery of the animals. The results of the antimicrobial susceptibility tests from the contract laboratory explained why the antimicrobials that have been administered before the susceptibility testing were inadequately effective. This circumstance emphasizes the importance of antimicrobial susceptibility testing before antibiotic treatment. In this study, neither *Enterococcus* spp. nor *E. coli* with antibiotic resistance were found in animal samples or in surrounding samples. The *Enterobacter* spp. isolates from hatchlings and chick paper all showed resistance to piperacillin, cefotaxime, ceftazidime, and mostly to fosfomycin (5 of 6 isolates) and intermediate reaction or resistance to ciprofloxacin (Figure 2). The results for *A. baumannii* from hatchlings or chick paper were very similar, with additional intermediate resistance toward temocillin, the combination of piperacillin/tazobactam, and amikacin. One isolate of *A. baumannii* from a drinking water pipe showed resistance to almost all tested antibiotics except levofloxacin and a combination of trimethoprim and sulfamethoxazole (Figure 2). Findings of extensively drug-resistant isolates are rare in farm animal samples and pose an alarming signal of extensive antibiotic usage. In the sprinkler system, the *P. aeruginosa* isolates showed resistance to cefotaxime, tigecycline, chloramphenicol, and fosfomycin and a reduced susceptibility toward a combination of trimethoprim and sulfamethoxazole. One *P. aeruginosa* isolate was additionally resistant to ciprofloxacin and levofloxacin. *Pseudomonas aeruginosa* isolates from drinking water lines showed increased diversity of antimicrobial resistance compared with that of the isolates from the sprinkler system (Figure 2). This finding is possibly caused by the administration of antimicrobials through the drinking water system, so antimicrobials acted as selectors for different resistances in the water lines. In addition, owing to the direct contact between drinking water lines and colonized animals, a direct exchange can occur, leading to adhesion of bacterial flora of the animals into the biofilm of drinking water pipes. This possibly explains differences in the resistance profile of bacteria from the sprinkler system and the drinking water lines. It seems obvious that the health problems of this farm were caused by 2 different factors. On the one hand, the chicks were already exposed to resistant bacteria, predominately *Enterobacter* spp., at the hatchery and arrived contaminated. On the other hand, *P. aeruginosa*–contaminated water was provided to the restocked chicks via the drinking lines and via mist by the sprinkler system from the first day in both barns. *Pseudomonas aeruginosa* is known to form biofilms in aqueous environments, such as water pipes and siphons in animal and human environments (Sib et al., 2019). They usually persist long term, causing recurring infections in chickens. Biofilm formation in water pipes is a recurring process, especially after restocking when water flow is low, as pipes offer good growth conditions for bacteria. Application of vitamin supplements, which are often mixed with glucose, and other medicinal treatments via water lines delivers sufficient nutrients for bacteria and biofilm formation. The temperature in broiler barns also enhances bacterial growth in water pipes (Maes et al., 2019). Infections with *P. aeruginosa* occur mostly from environmental contamination (Wingender and Fleming, 2011). On this farm, the continuous spraying of mist contaminated with resistant and susceptible *P. aeruginosa* caused a major health problem. Usually, *P. aeruginosa* are opportunistic pathogens that lead to secondary infections when the immune status of chickens is already depressed and can cause septicemia, skin lesion infections, and hemorrhagic pneumonia (Gong et al., 2018). Young birds are more susceptible than older birds to infection with *P. aeruginosa*, but infections can occur at any age (Gerlach, 1994; Joh et al., 2005). As a consequence of the findings at the first sampling, more specific hygiene measures, such as enhancing the exposure time for detergents and disinfectants in water lines or disassembling of the water filters, were implemented on the farm, as described above. The effect of the adopted measures could be seen by the results of the second sampling, where the analyzed ESBL and coliform bacteria were all below the detection limit of 1.0 log_10_ cfu ⋅ ml^−1^. Only in one sample from the sprinkler system was *P. aeruginosa* persistent. The results from this case study emphasize the importance of proper hygiene management to reduce antibiotic usage and the spread as well as the development of antibiotic resistance. The antibiotic resistance pattern that had already been acquired in the hatchery remains a major problem in fattening farms and needs to be addressed in future investigations (Projahn et al., 2016). Therefore, the aim should be to minimize the conscious use of antibiotics in broiler breeder farms to avoid early entry of resistance to the broiler meat production chain. Regarding extensively drug-resistant *A. baumannii*, further investigations on this farm should be carried out to prove whether this animal and human health–threatening strain was stably eliminated by sanitation improvements. In conclusion, systematic investigations by sampling not only the chicks but also the barns before restocking helps uncover critical points in hygiene and might be used as a basis for consultation or as a service of the cleaning contractors to successfully eliminate potential pathogens by implementing targeted measures.
